# Gene expression profiles of gliomas in formalin-fixed paraffin-embedded material

**DOI:** 10.1038/bjc.2011.547

**Published:** 2011-12-20

**Authors:** L A M Gravendeel, J J de Rooi, P H C Eilers, M J van den Bent, P A E Sillevis Smitt, P J French

**Affiliations:** 1 Department of Neurology, Erasmus Medical Center, Dr Molewaterplein 50, Rotterdam 3015 GE, The Netherlands; 2 Department of Bioinformatics, Erasmus Medical Center, Dr Molewaterplein 50, Rotterdam 3015 GE, The Netherlands; 3 Department of Biostatistics, Erasmus Medical Center, Dr Molewaterplein 50, Rotterdam 3015 GE, The Netherlands; 4 Department of Neuro-oncology, Daniel den Hoed Cancer Center, Groene Hilledijk 301, Rotterdam 3075 EA, The Netherlands

**Keywords:** glioma, expression profiling, FFPE, molecular clustering, exon array

## Abstract

**Background:**

We have recently demonstrated that expression profiling is a more accurate and objective method to classify gliomas than histology. Similar to most expression profiling studies, our experiments were performed using fresh frozen (FF) glioma samples whereas most archival samples are fixed in formalin and embedded in paraffin (FFPE). Identification of the same, expression-based intrinsic subtypes in FFPE-stored samples would enable validation of the prognostic value of these subtypes on these archival samples. In this study, we have therefore determined whether the intrinsic subtypes identified using FF material can be reproduced in FFPE-stored samples.

**Methods:**

We have performed expression profiling on 55 paired FF-FFPE glioma samples using HU133 plus 2.0 arrays (FF) and Exon 1.0 ST arrays (FFPE). The median time in paraffin of the FFPE samples was 14.1 years (range 6.6–26.4 years).

**Results:**

In general, the correlation between FF and FFPE expression in a single sample was poor. We then selected the most variable probe sets per gene (*n*=17 583), and of these, the 5000 most variable probe sets on FFPE expression profiles. This unsupervised selection resulted in a better concordance (*R*^2^=0.54) between expression of FF and FFPE samples. Importantly, this probe set selection resulted in a correct assignment of 87% of FFPE samples into one of seven intrinsic subtypes identified using FF samples. Assignment to the same molecular cluster as the paired FF tissue was not correlated to time in paraffin.

**Conclusion:**

We are the first to examine a large cohort of paired FF and FFPE samples. We show that expression data from FFPE material can be used to assign samples to intrinsic molecular subtypes identified using FF material. This assignment allows the use of archival material, including material derived from large-randomised clinical trials, to determine the predictive and/or prognostic value of ‘intrinsic glioma subtypes’ on Exon arrays. This would enable clinicians to provide patients with an objective and accurate diagnosis and prognosis, and a personalised treatment strategy.

The classification of tumour subtypes influences treatment decisions for many types of cancer. Accuracy in classifying cancer subtypes is therefore necessary to provide patients with correct diagnosis, prognosis and an optimal treatment strategy. As histological classification is often difficult in poorly differentiated tumours, this classification method urgently needs improvement. Gene expression profiling of cancer offers an accurate and objective method for classifying cancer subtypes (Sorlie *et al*, 2001; Valk *et al*, 2004). For example, in gliomas, the most common primary brain tumour in adults, gene expression profiling has identified distinct intrinsic subtypes of gliomas (Nutt *et al*, 2003; Freije *et al*, 2004; Phillips *et al*, 2006; Louis *et al*, 2007; Shirahata *et al*, 2007, 2009; Gravendeel *et al*, 2009; Li *et al*, 2009; Madhavan *et al*, 2009; Verhaak *et al*, 2010). We have performed unsupervised gene expression profiling in a cohort of 276 gliomas of all histological subtypes (Gravendeel *et al*, 2009). In this largest single-institution study conducted to date, we identified seven molecular glioma clusters. The molecular clusters were significantly better predictor of survival than histology, and were characterised by specific genetic changes. Data were validated and confirmed on six large external data sets. When validated in prospective studies these molecular clusters could contribute to clinical decision making. However, this study was conducted using RNA isolated from fresh frozen (FF) tissue. Unfortunately, FF tissue is scarce; most of the tissue archives with matched clinical outcome data are fixed in formalin and embedded in paraffin (FFPE). RNA isolated from FFPE material is often degraded and chemically modified as a result of the archiving method (Masuda *et al*, 1999; Farragher *et al*, 2008). However, new techniques have shown promising results in genome-wide expression profiling of RNA isolated from FFPE (Hoshida *et al*, 2008; Linton *et al*, 2008, 2009; Hall *et al*, 2011; Mittempergher *et al*, 2011).

Techniques used to study gene expression with FFPE material thus far have mostly been limited to single-gene analysis with RT–qPCR. Such techniques have demonstrated to be clinically relevant on a limited set of ‘classifier’ genes (Ma *et al*, 2006; Colman *et al*, 2010). Other multiplex assays (DASL, Quantigene, Nanostring, Fluidigm) have also shown promising results using distinct classifier genes (Canales *et al*, 2006; Geiss *et al*, 2008; Hoshida *et al*, 2008; Linton *et al*, 2008, 2009; Spurgeon *et al*, 2008; Hall *et al*, 2011; Mittempergher *et al*, 2011). Although whole-genome approaches for degraded RNA samples have improved over the last few years, the performance of current techniques to detect more subtle differences between cancer subtypes remains to be confirmed.

In this study, we therefore have performed expression analysis using a large cohort paired FF-FFPE glioma tissue using Exon 1.0 ST ‘exon’ arrays (Affymetrix, Santa Clara, CA, USA). Expression profiling using such a cohort has thus far not been performed. Most expression profiling studies using FF tissues have been performed on HU133-type arrays (A, A+B or the +2.0 version), whereas best results using FFPE samples have been obtained using the Exon 1.0 ST arrays. In this study, we therefore compare the expression of FF samples on HU133 plus 2.0 arrays with FFPE samples on Exon 1.0 ST arrays. Previous studies have demonstrated good overall correlation of HU133 Plus 2.0 with Exon 1.0 ST arrays (Okoniewski *et al*, 2007). We show that expression data from FFPE glioma material is concordant with expression data from matched FF tissue, and can be used for molecular profiling in gliomas. Furthermore, this molecular profiling is able to identify the subtle differences between the molecular glioma subtypes.

## Patients and methods

### Patient samples

We selected 55 paired FF-FFPE samples from the Erasmus University Medical Center glioma tumour archive. The FF and the FFPE samples were taken simultaneously from the tumour as parallel biopsies. All samples were visually inspected at the time of this study for the tumour content by the neuropathologist so that samples containing at least 80% of tumour tissue were selected. The FF samples selected were used in a previous study in which seven molecular clusters were identified (Gravendeel *et al*, 2009). The selection contained ∼10 samples from each molecular cluster. FF expression profiling results were reported previously (Gravendeel *et al*, 2009). The RNA from the FF tissue was extracted and hybridised in 2008. The RNA from the FFPE tissue was extracted and hybridised in 2010. Clinical and molecular data from the glioma samples included were reported previously (Gravendeel *et al*, 2009). The use of patient material was approved by the Institutional Review Board of the ErasmusMC, Rotterdam, the Netherlands (nr MEC 221.520/2002/262; date of approval 22 July 2003, and MEC-2005-057, date of approval 14 February 2005). For this use, patients gave written informed consent according to the Institutional and National guidelines. The fixation method of tissue in the Erasmus MC did not change over the last 25 years.

### RNA from FFPE extraction

Five sections of 10 *μ*m thick were cut from each tissue block. The High Pure RNA Paraffin Kit (Roche Applied Science, Mannheim, Germany) was used to isolate the RNA from the paraffin. After isolation the RNA was purified by ethanol precipitation (Supplementary Table 1). The quantity and integrity of the RNA was measured using a Nanodrop, and an Agilent 2100 BioAnalyzer RNA 6000 Nano Assay (Agilent Technologies, Amstelveen, the Netherlands). After total RNA isolation and purification, samples were diluted to 50 ng *μ*l^−1^ and stored at −80°C until use.

### qPCR

We randomly selected 11 samples that were assigned to two molecular clusters based on the FF expression data (*Cluster* 9 (*n*=5) and *Cluster 18* (*n*=6); Gravendeel *et al*, 2009). Four genes (two upregulated, two downregulated) that discriminate between the two subtypes were examined for differential gene expression (EMP3, SLC2A10, SUSD5 and CSMD3). These genes were identified using a *t*-test in combination with fold change. ACTB, GAPDH were used as control. All reactions were performed in duplicate. Primers and conditions are described in Supplementary Table 2.

### Arrays

A total of 150 ng per sample of the extracted RNA (FFPE) was used for the Exon 1.0 ST arrays (Affymetrix). Sample labelling and array hybridisation were performed by AROS Applied Biotechnology AS (Arhus, Denmark) according to the standard Affymetrix protocols in combination with Nugen WT-Ovation technology (FFPE V2 and Exon modules; San Carlos, CA, USA; *n*=55). Expression arrays (HU 133 plus 2.0 (Affymetrix) using FF material was reported previously (Gravendeel *et al*, 2009).

Quantile normalised robust multichip average (RMA) expression levels of 22 011 genes and 287 329 exons were extracted from Affymetrix Exon 1.0 ST arrays using Expression Console (Affymetrix). ClusterRepro (an R package) was used to assign a sample to a defined molecular subtype (Kapp and Tibshirani, 2007).

### Statistics

Differences between Kaplan–Meier survival curves were calculated by the log-rank (Mantel–Cox) test. Differences in age and RIN-scores of the tissue blocks were calculated using a *t*-test and a Mann–Whitney test. Significance of correlation coefficients was calculated using the *P*-value calculator for correlation coefficients (http://www.danielsoper.com/statcalc3).

## Results

### Sample characteristics

At total of 55 FFPE samples were included in the study. These samples included 29 glioblastomas, 5 astrocytomas grade III, 5 as grade II, 4 mixed oligoastrocytoma grade III (OA II), 2 OAs grade II, 8 oligodendrogliomas grade III and 2 pilocytic astrocytoma. The median time in paraffin of the FFPE samples was 14.1 years (range 6.6–26.4 years). The median RIN score of the RNA was 2.4 (range 1.1–2.7). Sample characteristics are listed in Table 1.

### qPCR

**Figure 1:**
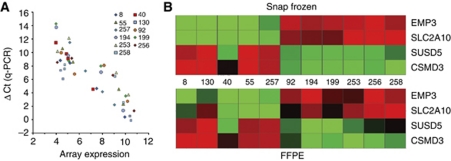
Correlation of the expression of the RNA (FFPE) and the RNA (FF) of 11 matched FF-FFPE samples. The four genes (EMP3, SLC2A10, SUSD5, CSMD3) chosen are the most discriminating genes between two very distinct molecular subtypes. (**A**) The correlation between the ΔCt of the qPCR results (FFPE material) and the expression data of the HU133plus 2.0 arrays (FF tissue). A high ΔCt value is indicative for a low expression value. The correlation plot shows a good correlation between the FF expression and the FFPE expression. (**B**) This correlation view shows the correlation of the expression of the FF tissue (HU133 plus 2.0 array) and the expression of the FFPE tissue (qPCR). The green color represents low expression, red represents high expression. The molecular clusters can be identified using the RNA isolated from FFPE. The color reproduction of this figure is available at the *British Journal of Cancer* online.

We first aimed to determine whether differences identified using expression profiling on snap frozen tissue could be found on RNA isolated from FFPE samples. For this initial test, we selected for samples that were assigned to two distinct molecular clusters based on the FF expression data (*Cluster* 9 (*n*=5) and *Cluster 18* (*n*=6); Gravendeel *et al*, 2009). *Cluster 9* shows a favourable prognosis compared with the other clusters, and is specific for loss of heterozygosity of 1p and 19q, as well as a high frequency of IDH1 mutations. *Cluster 18* has poor prognosis and is characterised by EGFR amplifications and CDKN2A deletions. The RT–qPCR results showed that both direction and fold change of all four genes in all samples could be recapitulated on RNA isolated from FFPE samples. The overall correlation was relatively strong *r*^2^=0.61 (*P*<0.001). Correlations (*r*^2^) and *P*-values for individual genes *EMP3*, *SUSD5*, *CSMD3* and *SLC2A10* were 0.34 (*P*=0.024), 0.840 (*P*<0.001), 0.849 (*P*<0.001) and 0.255 (*P*=0.047), respectively. These results demonstrate that differences in gene expression are retained in RNA isolated from FFPE samples (Figure 1).

### Exon array expression data and molecular clustering analysis

After the hybridisation of the exon 1.0 ST arrays, we compared the RMA normalised expression data of the exon arrays (FFPE) with exon 1.0 ST arrays that were analysed in earlier studies (FF tissue; French *et al*, 2007; Schutte *et al*, 2008). The exon arrays with FF tissue showed expression of more probe sets, as well as higher expression levels than the exon arrays with FFPE material (Supplementary Figure 2). On Hu133 plus 2.0 arrays (using FF samples), 9261±117 (52.9%) probe sets are expressed at RMA levels >6.5. On exon arrays, 59 618±20 337 (20.7%) probe sets are detected at *P*<0.01, using DABG values. It should be noted that significantly more probe sets are detected on exon arrays using FF tissue (141 493±12 924 (49.2%), see also Supplementary Figure 1). In addition, the distribution of the RMA expression histograms of the FFPE glioma tissue is shifted compared with the expression histograms of exon arrays with FF tissue (Supplementary Figure 3).

### Correlation expression FF *vs* FFPE

Exon arrays contain one or more probe sets per exon for each gene (287 329 core probe sets for 22 011 genes), whereas only one data point per gene is generated on HU133plus2 arrays (17 583 genes, when using the alternative .cdf based on entrezgene; Dai *et al*, 2005). We therefore first selected the probe sets on exon arrays that likely contain most of the biological information. Because genes that discriminate between molecular subtypes are by definition differentially expressed, and thus show a relatively high variance in expression, we selected the probe set with highest variance per gene (*n*=17 583 probe sets (6.2% of all ‘core’ probe sets); log2 normalised data). Selecting the most variable probe set on exon arrays does not always identify those with highest correlation to expression on HU133plus2.0 arrays. However, selection based on variance approaches both data sets independently and avoids potential circular arguments. All further analysis was therefore done using the selection of exon array probe sets based on variance as starting data set.

**Figure 2:**
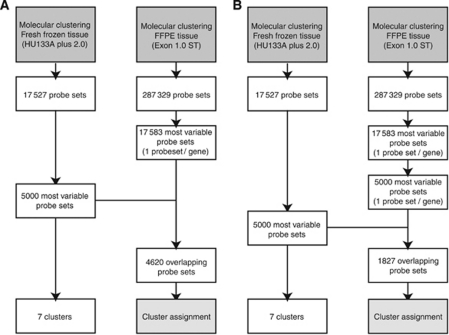
Flow charts of the selection of probe sets containing the most informative gene expression data. (**A**) We first selected the probe set with highest variance per gene (*n*=17 583 probe sets; FFPE: 17 583). In our previous study, we performed molecular clustering with FF tissue based on the 5000 most variable genes (FF: 5000). The overlap between the most variable probe sets in FF tissue and the most variable probe sets in FFPE tissue (FF: 5000/FFPE: 17 853) consisted of 4620 matching probe sets. On the basis of these 4620 probe sets, samples were assigned to one of the seven molecular clusters using ClusterRepro. (**B**) When using exon arrays, it is possible that no informative probe sets are available for a single gene, and such probe sets may be filtered out by selecting not only the most variable probe set per gene, but also, of these, the most variable 5000 probe sets (FF: 5000/FFPE: 5000). By using this filter, there are 1827 overlapping probe sets. ClusterRepro was used to assign the samples to one of the seven molecular clusters based upon these overlapping probe sets.

In our previous study, we performed molecular clustering with FF tissue based on the 5000 most variable genes (FF: 5000). The overlap between the most variable probe sets in FF tissue and the most variable probe sets in FFPE tissue (FF: 5000/FFPE: 17 853) consisted of 4620 matching probe sets (Figure 2A).

The set of 17 853 probe sets assumes that all genes have at least one informative probe set per gene. It is however possible all probe sets that belong to the same gene perform poorly. We therefore also performed a further selection of the 17 583 exon array probe sets, by selecting the 5000 most variable probe sets of these 17 583 (1.74% of the total number of ‘core’ probe sets, Figure 2B). Using the 5000 most variable probe sets for both FF and FFPE material showed an overlap of 1827 matching probe sets (FF: 5000/FFPE: 5000, 1827 overlapping probe sets; Figure 2B).

**Figure 3:**
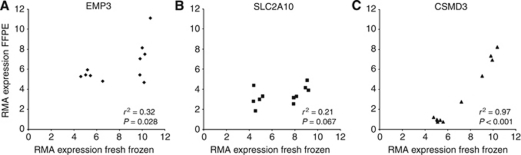
Correlation of the qPCR genes (EMP3, CSMD3 and SLC2A10) between the expression of the FF RNA and the FFPE RNA. We compared the gene expression data (FF; HU133 plus 2.0) of the four genes used in the qPCR analysis (EMP3, SUSD5, CSMD3 and SLC2A10) with the expression data of the FFPE material (Exon 1.0 ST). We used the expression data of the same 11 samples that were also used in the qPCR analysis. (**A**) and (**B**) show a weak correlation for both EMP3 (*r*^2^=0.32) SLC2A10 (*r*^2^=0.21). A good correlation is seen for CSMD3 (*r*^2^=0.97) (**C**). There were no data available of SUSD5 as there were no probe sets of this gene on exon 1.0 ST arrays.

We first compared the gene expression data (FF) of the genes used in the qPCR analysis (EMP3, SUSD5, CSMD3 and SLC2A10) with the expression data of the FFPE material. For this analysis we used the expression data of the same 11 samples that were also used in the qPCR analysis. Figure 3 shows the correlation of the qPCR genes (EMP3, CSMD3 and SLC2A10) between the expression of the FF RNA and the FFPE RNA. These results show a weak correlation between the FF and FFPE expressions for both EMP3 and SLC2A10 (*r*^2^=0.32; *P*=0.028 and *r*^2^=0.21; *P*=0.067), and a good correlation for CSMD3 (*r*^2^=0.97; *P*<0.001). There were no data available of SUSD5 as there were no probe sets of this gene on exon 1.0 ST arrays. These results highlight that exon arrays can show a concordance in gene expression compared with FF tissue, but that a selection of the biologically most informative probe sets is required.

We next compared the normalised expression data of the 5000 most variable genes as used in our previous study, with the expression of the most variable exons (FF: 5000/FFPE: 17 583, 4620 matching probe sets; Gravendeel *et al*, 2009). In general, the strength of correlation between FF and FFPE expression in a single sample (sample 8) was weak (*r*^2^=0.24, *P*<0.001). It should be noted that part of the between FF and FFPE sample variability is biological: The snap frozen and FFPE tissues are not taken from exactly the same location within a tumour. For this analysis we compared differential gene expression between samples of cluster 9 and 18 (separately for FF and FFPE). The correlation (*r*^2^) in differential gene expression between FF and FFPE was 0.38.

**Figure 4:**
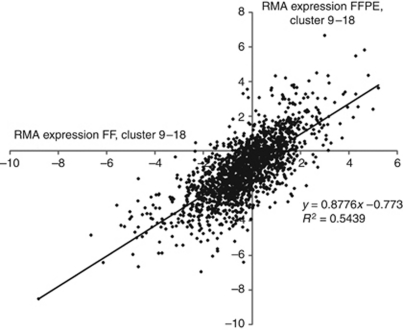
Differential gene expression between samples of *Cluster 9* and *18*. The differential gene expression between samples of two molecularly very distinct clusters (*Cluster 9* and *18*) showed a relatively good correlation between 18 FF samples on HU133 plus 2.0 and 18 FFPE samples on HuEx 1.0 ST arrays (*R*^2^=0.54). For this analysis we used the 5000 most variable probe sets of the exon expression data and subtracted the median expression of *Cluster 18* from the median expression of *Cluster 9*.

We did the same analysis for the most variable 5000 probe sets (FF: 5000/FFPE: 5000, 1827 overlapping probe sets). Differential gene expression between samples of *Cluster 9* and *Cluster 18* then showed a relatively strong correlation between FF on HU133 plus 2.0 and FFPE on HuEx 1.0 ST arrays (*R*^2^=0.54; *P*<0.001 (Figure 4). Our results demonstrate that differential gene expression between samples observed using RNA isolated from FF tissue is at least partially retained on RNA isolated from FFPE samples.

Expression data of the FF samples is performed HU133 Plus 2.0 arrays, a platform that is different from the platform used for the FFPE samples (HuEx 1.0 st arrays). We have therefore compared expression of all probes (287 329) between FF and FFPE on exon arrays of eight matched samples (8, 40, 130, 206, 257, 259, 275 and 293). In general, the correlation between FF and FFPE samples on the same platform was reasonable (*r*^2^=0.315±0.093 range 0.210–0.450, *P*<0.001). This correlation was much better than the overall correlation (also using 287 329 probe sets) between FF on HU133 Plus 2.0 arrays and FFPE on HuEx 1.0 st arrays (0.034±0.023). The better correlation between FF and FFPE samples on the same platform therefore indicates that differential gene expression is better retained when using the same platform.

### Cluster assignment

Recently, we described the identification of seven molecular glioma subtypes based on gene expression profiling, which are better predictors of survival than histology (Gravendeel *et al*, 2009). Our final assessment to determine the suitability of RNA isolated from the FFPE samples was to confirm sample assignment to individual molecular subtypes. Clustering results are represented in Table 2. Overall, assignment to the correct cluster (e.g., assignment to the same molecular cluster as the FF tissue in the previous study) was seen in 76% (*n*=42) of the samples (FF: 5000/FFPE: 17 583). However, part of the variability between FF and FFPE samples is biological and may represent tumour heterogeneity. This heterogeneity is specifically notable for assignment to *Cluster 0*, as assignment to this cluster depends on the relative amount of non-neoplastic tissue present. Indeed, the overlap between FF and FFPE cluster assignment when excluding samples that are assigned to *Cluster 0* is 86% (*n*=42/49). A total of 13 samples were assigned to a different molecular cluster, 7 without *Cluster 0*.

Similar performance in cluster assignment was observed when using the 5000 most variable exon probe sets (FF: 5000/FFPE: 5000; Figure 2B). Assignment to the identical cluster was seen in 75% (*n*=41) of the samples, 87% without *Cluster 0* (*n*=41/47).

Assignment to the same molecular cluster as the paired FF tissue did not have a significant correlation with the time in paraffin. The ‘wrongly’ assigned blocks even showed a slightly shorter median time in paraffin than the ‘correctly’ assigned samples (11.9 *vs* 14.3 years; *P*=0.07). The average RIN score of the incorrectly assigned samples was 2.18±0.40, and was not significantly different from the RIN scores of the correctly assigned samples 2.24±0.34, *P*=0.71).

**Figure 5:**
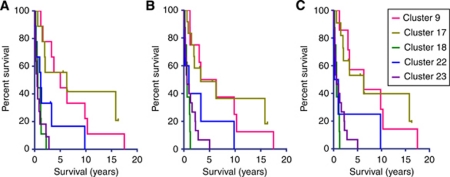
Kaplan–Meier survival curves of the seven molecular clusters identified in FF and FFPE material show a high resemblance. (**A**) shows the survival of the seven molecular clusters identified, using FF material of the 55 patients included in this study (HU133 plus 2.0 arrays). (**B**) shows the survival curves of the molecular clusters to which the FFPE tissue was assigned based on the 17 852 most variable probe sets from the Exon 1.0 ST arrays. (**C**) Shows the survival curves of the molecular clusters to which the FFPE tissue was assigned by filtering the exon data on the 5000 most variable probe sets.

The high degree of overlap between FF and FFPE sample assignment (both FF: 5000/FFPE: 17 583 and FF: 5000/FFPE: 5000) is reflected in a highly similar patient survival curves (Figure 5). However, FFPE survival curves also include three samples that originally were assigned to *Cluster 0*.

## Discussion

In this study, we describe a method that allows analysis of gene expression profiling of FFPE cancer tissue using HuEx 1.0 ST arrays. Our data show that expression data of RNA isolated from FFPE and FF tissues are comparable. However, a selection on the most informative probe sets (based on highest variance for each probe set/gene and highest variance between genes) is required. RIN score and the age of the FFPE tissue blocks do not influence the gene expression results. The average RIN score and the average time in paraffin of the incorrectly assigned samples were not significantly different from the RIN scores and time in paraffin of the correctly assigned samples. The probe sets identified in this study can be used in other profiling studies that lack paired FF samples.

Differential gene expression between samples is well retained (Figure 4). This is also illustrated by the identical assignment to intrinsic molecular subtypes for both FF and FFPE glioma tissue in up to 87% of the samples (Table 2). It should be noted that FF and FFPE tissues are resected from different parts of the tumour. Therefore, tumour heterogeneity may also contribute to the differential assignment between FF and FFPE samples.

Previous studies demonstrated that FFPE samples can be used for gene expression profiling either using the Affymetrix (Exon 1.0 and HU133 plus 2.0) or using the Illumina (DASL) platforms (Hoshida *et al*, 2008; Linton *et al*, 2008, 2009; Hall *et al*, 2011; Mittempergher *et al*, 2011). However, these studies had limited sample size, lacked controlled experiments with paired FF-FFPE sample analysis or were used to differentiate between very distinct cancers. Our study is the first to use a large cohort of paired FF-FFPE glioma samples for expression profiling with exon 1.0 ST arrays. We show that degraded RNA that is up to 25 years old, is suitable to identify subtle differences between subtypes within one specific cancer.

Other genome-wide techniques are also available that can perform expression profiling on FFPE samples, including the DASL platform (Illumina). The platform chosen for this study was based on reports from literature (Linton *et al*, 2008, 2009), and it is beyond the scope of this manuscript to compare performance of both platforms. Although it is possible other platforms perform better on FFPE samples, our study demonstrates that sufficient information is stored in FFPE samples so that it can be used for expression profiling using exon 1.0 ST arrays. Our method allows molecular classification of archived clinical trial samples to evaluate the predictive and prognostic values of the molecular glioma clusters. Furthermore, it allows assignment of FFPE material of newly diagnosed patients to molecular clusters. Such assignment would allow clinicians to improve patients’ diagnosis and would contribute to treatment decisions.

## Figures and Tables

**Table 1:** Patient and sample characteristics

**Db no**	**Survival (years)**	**Status**	**PA diagnosis**	**Age FFPE material (years)**	**RIN score**	**Correlation (*r*^2^) FF and FFPE**	***P*-value correlation FF and FFPE**
8	9.82	Dead	OD III	15.3	1.90	0.24	<0.001
40	10.28	Dead	OD III	18.9	2.20	0.29	<0.001
55	17.49	Dead	OD III	23.9	2.30	0.30	<0.001
63	3.28	Dead	GBM	9.9	2.40	0.12	<0.001
77	1.30	Dead	GBM	12.2	2.60	0.03	0.02
92	1.26	Dead	GBM	11.3	1.10	0.20	<0.001
98	0.98	Dead	GBM	12.1	2.30	0.13	<0.001
99	0.86	Dead	GBM	13.3	N/A	0.03	0.024
104	0.21	Dead	GBM	10.4	N/A	0.15	0.024
105	1.03	Dead	GBM	16.0	N/A	0.08	<0.001
112	0.59	Dead	GBM	16.0	N/A	0.10	<0.001
119	0.18	Dead	GBM	13.1	2.30	0.15	<0.001
123	2.05	Dead	GBM	13.9	2.50	0.15	<0.001
130	6.31	Dead	GBM	15.1	1.40	0.08	<0.001
134	1.48	Dead	A II	13.9	N/A	0.06	<0.001
143	4.79	Dead	A II	13.4	2.70	0.10	<0.001
174	0.28	Dead	GBM	19.3	N/A	0.05	<0.001
183	0.12	Dead	GBM	12.3	N/A	0.14	<0.001
194	0.19	Dead	A III	20.8	1.90	0.13	<0.001
198	6.27	Dead	A III	21.8	2.40	0.11	<0.001
199	1.11	Dead	GBM	20.2	2.10	0.14	<0.001
206	0.29	Dead	GBM	11.7	2.50	0.03	0.028
209	1.96	Dead	OA III	11.0	2.50	0.09	<0.001
227	3.10	Lost to follow-up	GBM	10.9	N/A	0.01	0.22
253	0.62	Dead	GBM	9.3	2.20	0.32	<0.001
256	0.27	Dead	GBM	14.1	2.40	0.15	<0.001
257	3.30	Dead	OD III	17.9	2.50	0.23	<0.001
258	2.26	Dead	GBM	18.4	2.50	0.12	<0.001
259	5.02	Dead	OD III	13.5	2.40	0.02	0.087
286	5.56	Lost to follow-up	GBM	24.6	2.30	0.13	<0.001
291	0.63	Dead	OA III	20.3	2.10	0.24	<0.001
293	2.99	Dead	OD III	22.7	2.50	0.10	<0.001
315	0.61	Dead	GBM	15.4	N/A	0.14	<0.001
336	1.19	Dead	A II	11.9	2.40	0.15	<0.001
353	9.79	Dead	GBM	26.0	2.10	0.10	<0.001
380	1.61	Dead	GBM	7.5	2.20	0.04	0.002
387	3.32	Dead	GBM	17.0	N/A	0.12	<0.001
393	0.06	Dead	GBM	14.2	2.10	0.17	<0.001
416	15.82	Dead	OD III	26.4	2.50	0.14	<0.001
420	1.32	Dead	A II	17.4	N/A	0.06	<0.001
441	3.76	Dead	OA II	18.9	2.50	0.02	0.06
445	1.18	Dead	OA III	18.9	2.20	0.25	<0.001
446	0.45	Dead	A III	8.9	1.60	0.04	<0.001
467	0.05	Dead	A III	10.9	2.50	0.01	0.15
473	1.20	Dead	A III	22.7	2.50	0.20	<0.001
515	0.35	Dead	GBM	7.4	2.50	0.00	0.45
536	6.39	Alive	OA II	6.6	2.40	0.13	<0.001
565	2.79	Dead	GBM	14.2	1.90	0.23	<0.001
566	0.48	Dead	GBM	13.8	N/A	0.18	<0.001
568	16.31	Alive	OA III	16.5	2.60	0.20	<0.001
619	0.48	Dead	OD III	11.9	2.50	0.28	<0.001
628	7.52	Dead	A II	10.9	N/A	0.21	<0.001
629	2.22	Dead	GBM	10.8	2.50	0.08	<0.001
711	14.18	Alive	PA	14.3	1.30	0.10	<0.001
712	0.19	Lost to follow-up	PA	13.9	2.60	0.17	<0.001

Abbreviations: A III=astrocytoma grade III; A II=astrocytoma grade II; Db=database number; FF=fresh frozen; FFPE=fixed in formalin and embedded in paraffin; GBM=glioblastoma; OA II=oligoastrocytoma grade II; OA III=oligoastrocytoma grade III; OD II; oligodendroglioma grade II; OD III=oligodendroglioma grade III; RIN=RNA integrity number.

**Table 2:** Cluster assignment of samples

**Db no**	**Clustering RNA (FF)**	**Clustering RNA FFPE 17 853 most variable exons**	**Clustering RNA FFPE 5000 most variable exons**
8	9	9	9
40	9	9	9
55	9	9	9
63	0	17	0
77	0	23	23
92	18	18	18
98	22	22	22
99	23	17	0
104	23	23	23
105	23	23	23
112	23	23	23
119	22	22	22
123	17	17	17
130	9	9	9
134	17	17	17
143	0	0	0
174	23	23	23
183	23	23	23
194	18	18	18
198	17	17	17
199	18	18	18
206	22	0	0
209	17	17	17
227	22	17	17
253	18	18	18
256	18	18	18
257	9	9	9
258	18	23	23
259	9	23	23
286	17	17	17
291	18	18	18
293	0	9	9
315	17	17	17
336	22	9	0
353	22	22	22
380	9	23	23
387	22	22	17
393	23	23	23
416	17	17	17
420	22	0	0
441	9	0	0
445	18	18	18
446	16	23	23
467	22	22	22
473	9	9	9
515	23	23	23
536	17	17	17
565	23	23	23
566	23	23	23
568	17	17	17
619	18	18	18
628	0	0	0
629	23	23	23
711	16	16	0
712	16	16	16

Abbreviations: FF=fresh frozen; FFPE=fixed in formalin and embedded in paraffin.
