# Supplementary figures and images for: Gene expression profiles of gliomas in formalin-fixed paraffin-embedded material
Source: Br J Cancer. 2011 Dec 20;106(3):538–45. doi: 10.1038/bjc.2011.547 (PMC3273349; doi:10.1038/bjc.2011.547)

## Slide 1
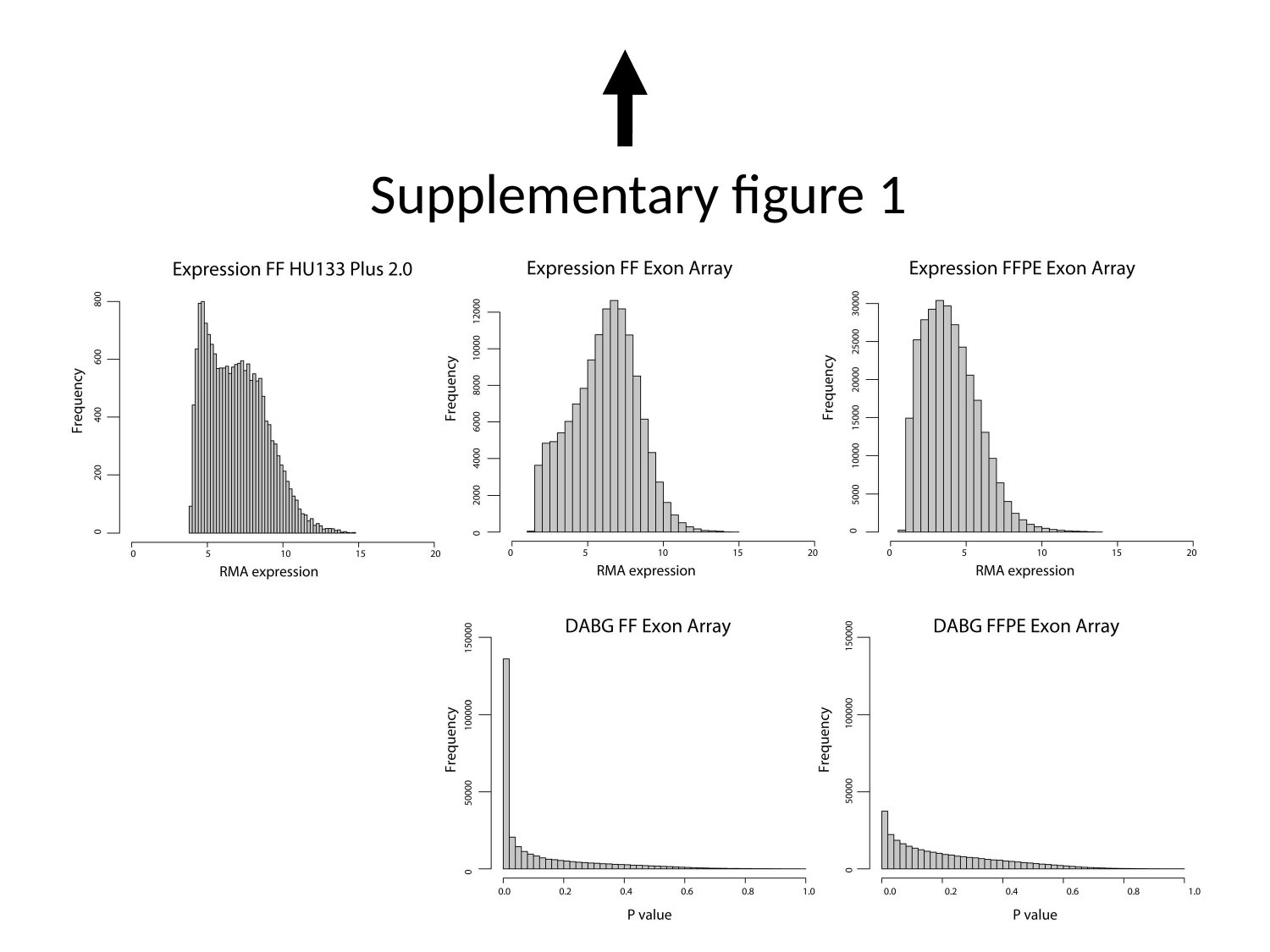

# Supplementary figure 1

## Slide 2
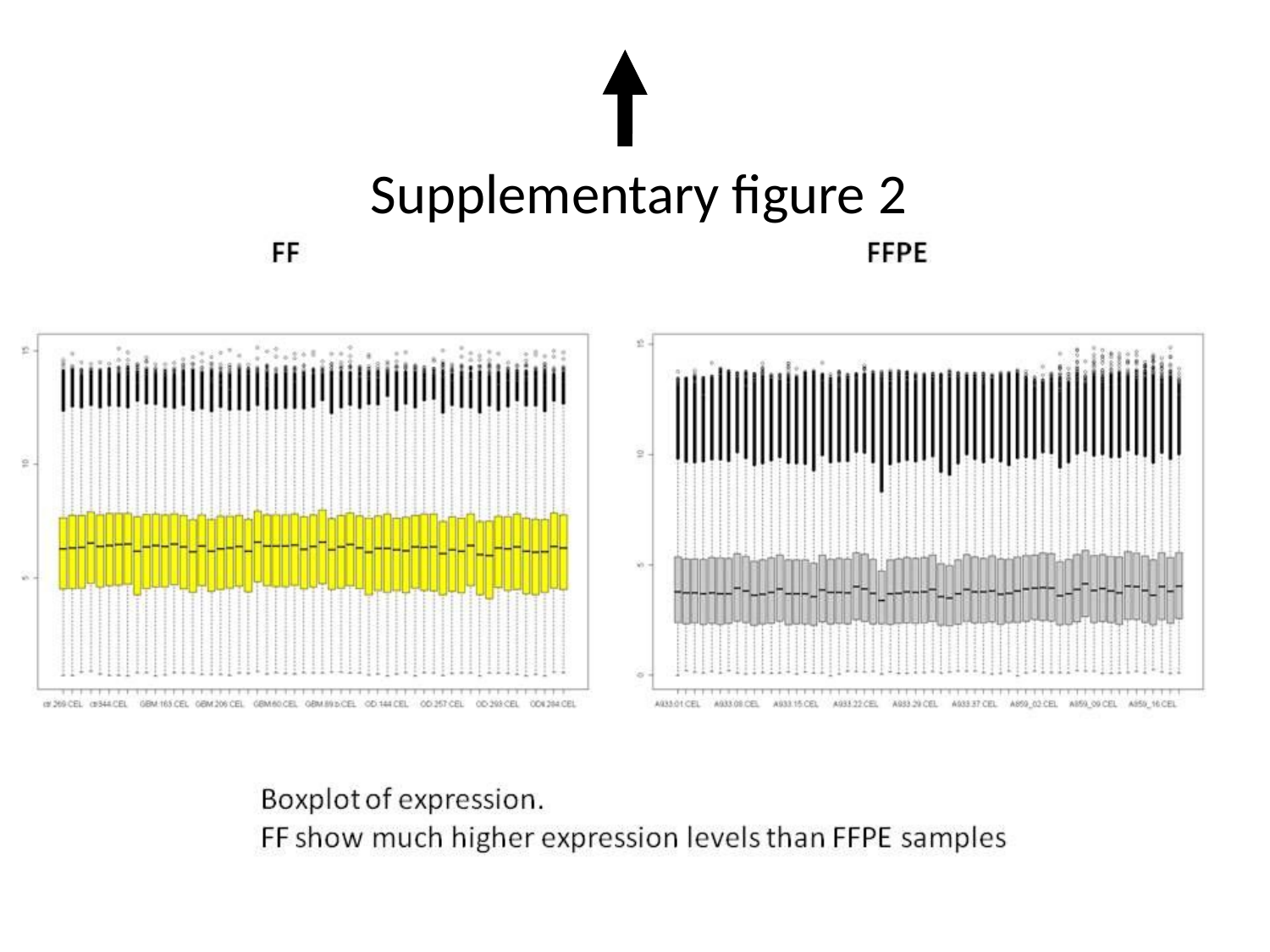

# Supplementary figure 2

## Slide 3
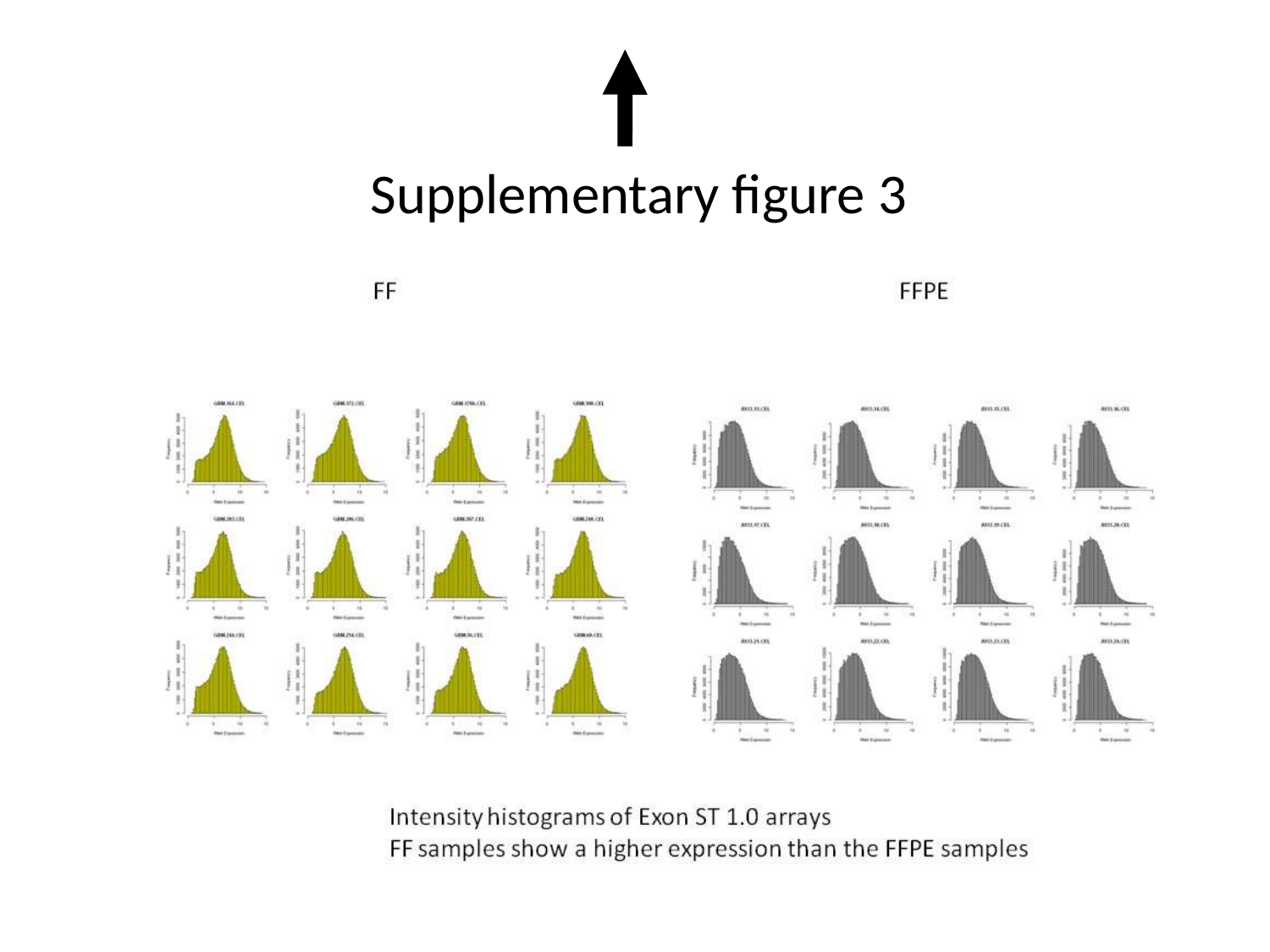

# Supplementary figure 3

Supplement: Supplementary Figures 1-3 [file bjc2011547x1.ppt]
