# Supplementary material for: Gene expression profiles of gliomas in formalin-fixed paraffin-embedded material
Source: Br J Cancer. 2011 Dec 20;106(3):538–45. doi: 10.1038/bjc.2011.547 (PMC3273349; doi:10.1038/bjc.2011.547)
Supplement: Supplementary Figure legends [file bjc2011547x2.doc]

**Supplementary figure legends**

**Supplementary figure 1**

**Comparison of expression and DABG values of samples run on HU133 Plus 2.0 arrays (FF, left), HU_Ex_1.0 st arrays (FF, middle) and HU_Ex_1.0 st arrays (FFPE, right).** Samples run with FF tissue showed a higher expression levels than samples run using FFPE material (top panels). In addition, more probesets are detected using FF material than FFPE material on HU_Ex_1.0 st arrays (bottom panels)

**Supplementary figure 2**

**Boxplot of expression values of FF and FFPE samples on HU_Ex_1.0 st arrays.** Samples run on exon arrays run using FF tissue showed a higher expression level of probesets than those run using FFPE material

**Supplementary figure 3**

**Intensity histograms of samples run on HU_Ex_1.0 st arrays.** The distribution of RMA expression histograms of the FFPE glioma tissue is shifted compared to the expression histograms of exon arrays with FF tissue.
