# Supplementary material for: Gene expression profiles of gliomas in formalin-fixed paraffin-embedded material
Source: Br J Cancer. 2011 Dec 20;106(3):538–45. doi: 10.1038/bjc.2011.547 (PMC3273349; doi:10.1038/bjc.2011.547)
Supplement: Supplementary Table 1 [file bjc2011547x3.doc]

**Suppplementary table 1**

| Ethanol precipitation  ·         50 µl sample  ·         Add 5 µl NaAC  ·         Add 3 µl glycogen  ·         Add 125 µl ethanol 100%  ·         Store at -20°C for 1 hour  ·         Centrifuge for 20 minutes, 13000 g, 4°C  ·         Discard supernatant  ·         Add 150 µl ethanol 80%  ·         Centrifuge for 5 minutes, 13000 g, 4°C  ·         Discard supernatant, leave pellet to dry for minutes  ·         Add 25 µl nuclease-free H2O  ·         Store at -80°C | | |  |  |  |  |
| --- | --- | --- | --- | --- | --- | --- |
|  |  |  |  |  |  |  |
|  | | |  |  |  |  |
|  | | |  |  |  |  |
|  | | |  |  |  |  |
|  | | | |  |  |  |
|  | | | |  |  |  |
|  | | | | |  |  |
|  | | | |  |  |  |
|  | | | |  |  |  |
|  | | | | |  |  |
|  | | | | | |  |
|  | | | |  |  |  |
|  | | |  |  |  |  |
|  |  |  |  |  |  |  |
|  |  |  |  |  |  |  |
